# Supplementary material for: Global hypo-methylation in a proportion of glioblastoma enriched for an astrocytic signature is associated with increased invasion and altered immune landscape
Source: eLife. 2022 Nov 22;11:e77335. doi: 10.7554/eLife.77335 (PMC9681209; doi:10.7554/eLife.77335)
Supplement: Figure 2—source data 1. [file elife-77335-fig2-data1.zip › Figure_2_source_data_1/Figure_2B/homerResults/motif14.similar.html]

motif14

## Information for motif14

C
T
A
G
T
A
C
G
C
T
G
A
T
A
C
G
T
A
G
C
G
A
C
T
T
C
A
G
C
A
G
T
C
T
A
G
T
C
A
G
C
T
G
A
T
A
C
G
C
T
G
A
C
T
A
G
T
C
A
G
  
Reverse Opposite:  

A
G
T
C
G
A
T
C
G
A
C
T
A
T
G
C
G
A
C
T
A
G
T
C
G
A
T
C
G
T
C
A
A
G
T
C
C
T
G
A
A
T
C
G
A
T
G
C
G
A
C
T
A
T
G
C
G
A
T
C
  

|  |  |
| --- | --- |
| p-value: | 1e-60 |
| log p-value: | -1.402e+02 |
| Information Content per bp: | 1.743 |
| Number of Target Sequences with motif | 109.0 |
| Percentage of Target Sequences with motif | 4.72% |
| Number of Background Sequences with motif | 2.8 |
| Percentage of Background Sequences with motif | 0.79% |
| Average Position of motif in Targets | 100.9 +/- 54.1bp |
| Average Position of motif in Background | 111.1 +/- 62.5bp |
| Strand Bias (log2 ratio + to - strand density) | -0.0 |
| Multiplicity (# of sites on avg that occur together) | 1.16 |
| Motif File: | file (matrix) reverse opposite |

### Similar de novo motifs found

|  |  |  |  |  |  |  |  |
| --- | --- | --- | --- | --- | --- | --- | --- |
| Rank | Match Score | Redundant Motif | P-value | log P-value | % of Targets | % of Background | Motif file |
| 1 | 0.613 | T C A G C A T G C A T G A C T G A C G T A T G C G C A T A G T C G A C T A T C G C T A G C T A G T A C G T G C A C T A G | 1e-54 | -125.927538 | 3.38% | 0.33% | motif file (matrix) |
| 2 | 0.667 | A G T C A G T C A C G T A G T C A C T G A G T C A G T C A G T C A G T C A G T C A C T G A G T C | 1e-30 | -69.181122 | 3.16% | 0.63% | motif file (matrix) |
